# Abstracts and Gleanings

**Published:** 1885-09-20

**Authors:** 


### ABSTRACTS AND GLEANINGS.

**The Communicability of Scarlet Fever.**—Dr. Mitchell, in Medical and Surgical Reporter, writes: I noticed an article in the last number of the Reporter, on "The Communicability of Scarlet Fever through a Third Healthy Person," and thinking my experience may add something to the literature of the subject, I write this hurried note. In the fall of 1878 Mrs. G. of this place, visited friends some fifteen miles or more from here, staying two or three weeks.

Previous to that time there had been no case of scarlet fever in this neighborhood, and no epidemics of the disease for several years. There were some few cases in the community where Mrs. G. was visiting, but, as subsequently learned, not seemingly of a malignant character. In about a week or ten days after her return, two of her little grandchildren were taken with the disease in so mild a form that, if it had not been for the throat-trouble and the peculiarly pasty condition of the tongue, it would hardly have been recognized as scarlet fever. The cases progressed favorably and the patients were soon well.

In the meantime, a family living about one or two hundred yards away from Mrs. G., and whom she visited soon after her return, were taken with the disease, in all its virulence, one of the children dying in a few days, and some five or six others had the disease with its unpleasant sequelæ, and tardy convalescence. These children were never outside their own door or yard, and the only possible communication was as above mentioned. In addition to this, my own little boy, as in the case of Dr. Hewetsen's, climbed on my lap after my return from seeing Mrs. G.'s grandchildren, and in the usual time the disease developed, and the child died in a few days. My other children also had it, but recovered, and the disease became general, spreading far and wide throughout the community.

In the fall of 1879, when there had been no known cases of scarlet fever for six months at least, a woman came on a visit to a family in another part of my field of practice, from a place some thirty or forty miles away, where scarlet fever was prevalent. The usual result followed in the family she was visiting. Every child in the family—seven—being attacked by malignant scarlet fever. All recovered, however. The disease spread, and was of the most malignant type I ever saw. One bright little fellow died in less than ten hours after he was taken, or at least after he complained of being sick, another in eighteen hours and so on. I hope to be spared from ever passing through epidemics of scarlet fever such as I have mentioned.

It seemed to spare neither age, sex nor "previous condition of servitude." I saw cases that were well-marked in persons as old as sixty years and more, and all ages younger than that.

I never knew a nursing child to die of scarlet fever, and regard the danger, other things being equal, as slight, even when malignant, after the age of puberty. Parturients, however, shoud be excepted. Since that time I have never had a doubt in regard to the communicability of scarlet fever.

In this connection, it may not be out of place for me to give a short similar testimony in regard to diptheria. Mr. M. and his wife, parents of a large family, all of them at home, except the two oldest daughters, married, went on a visit to one daughter, married, living not far from Wheeling, West Virginia, taking their youngest child, a boy some four or five years old with them. Whilst there he contracted diphtheria, which was prevailing there, and died after a short illness. The heart-broken parents buried their baby among strangers and returned home.

There had not been a case of diphtheria in this country for a long time, although scarlet fever was epidemic, but in a week or two diphtheria, in its most maglignant form, broke out in Mr. M.'s family, and four out of six remaining at home died. Mr. and Mrs. M. said they brought nothing with them that had been about the first child that died, so that the disease must have been carried in their own clothing.

**Carbuncle Treated with Oleate of Morphia.**—Dr. Hibberd, in Indiana Medical Society, reported two cases of carbuncle as follows: On the 30th of April, 1884, T. N. an active business man of general good health, about sixty years old, applied to me for a painful swelling on his neck to the left of the ligamentum nuchæ, a little below the line of hair.

Telling my patient that he had carbuncle, and was likely to have several weeks of great local suffering and much general depression, I gave him some oleate of morphia, with careful instructions how to apply it, expressing the hope that it would do something to mollify the pain if nothing more, and this I did because of my experience in alleviating other painful conditions of the skin and subcutaneous tissue with this preparation of morphia. It was also to be applied to the nodules forming near the principal swelling. The patient returned the next day, and quite to my surprise and gratification, stated that nearly all pain had ceased in the large tumor, all was gone from the smaller ones, and the soreness and stiffness of the neck had greatly diminished. On examination the smaller tumors were shrunken and no longer irritable, and the larger one had lost something of its boggy feel, was apparently smaller, was but slightly sore, and the skin over it was more natural in appearance. The application was continued, and at the end of three days all pain and tenderness had left the neck, the smaller tumors disappeared, and the larger ones had the characteristics of a calloused indurated swelling under the skin, about half the dimensions of the original tumor, and this was absorbed in about a week or more.

Two or three months subsequently the patient had another disturbance near the same spot, beginning in an irritable pimple as the former one had begun, but a few applications of the same remedy applied by my direction arrested all further development.

On the 24th of April, 1885, Mr. J. W. G., aged eighty-six years, sought my advise for a tumor on the back of his neck, to the right of the ligamentum nuchæ, which was giving him much pain and anxiety. It proved to be a carbuncle an inch and a half in diameter, with a point of superficial suppuration on top. An inch below the main tumor was a smaller one, an irritable pimple such as the larger one was in the beginning.

The swelling was of several days standing, and a part of the skin covering it so inflamed that I feared the oleate of morphia could not be used with the success that otherwise I should have hoped for. However, I gave him the preparation, with instructions for its diligent and proper use, but explained to him why it might fail, and advised him, if he had no relief after a fair trial, to consult another practitioner, as I should leave for New Orleans on the afternoon of the next day. But at noon the next day the old gentleman reappeared at my office with a smiling countenance to give me some good news, as he averred, before I left, stating that the soreness of the tumor was nearly gone, the stiffness of his neck but trifling, and the pain so promptly relieved that he had had a good night's rest, the first of the kind since the swelling began. On examination the soreness of the smaller tumor was removed, and that of the larger one greatly diminished, and its appearance and feeling altered for the better, while the superficial point of suppuration was discharging but little and the surrounding inflammation of the skin greatly subdued. Treatment was directed to be continued, and when I returned and examined the seat of disease ten days later, there still remained a small indurated nodule under the skin, but no soreness, no pain, nor other inconvenience, and to this condition it had been steadily approaching since my last previous examination.

**Chloroform Water.**—In Vol. CXII, No. 4, Boston Medical and Surgical Journal, chloroform water—water saturated with chloroform—is treated editorially. The writer, in describing it, says that it was first formularized by Guillot, in 1884, and that afterward it was made the subject of a series of trials by Lasegne, Reynauld, and more recently, by Beurmann. It is a stable preparation, easily prepared, and agreeable to the taste; and when diluted one-half with water it is devoid of all piquancy and acridity. After reading and studying the above named article on the subject, I immediately prepared some and began its use, substituting it for syrups in cough mixtures, and using it in all solutions containing iron. Besides its other merits, it has marked analgesic power. It is an admirable remedy in nausea, vomiting, and gastralgia, and with morphia it is one of the most desirable of sedative cough mixtures. It is said to disguise almost entirely the taste of salicylate of soda, chloral, and bromide of potassium. I have used several gallons of it, and I am daily more pleased with it. For a long time I have been disgusted with syrup mixtures (and I believe my patients have been also), and I shall use chloroform water in their place whenever I can.

To prepare it: Take a half gallon bottle and nearly fill it with distilled water; then add three or four fluid drachms of Squibbs' chloroform; cork it tightly, and shake it every five minutes for an hour or so, and then set it to one side until the excess of chloroform has settled at the bottom of the bottle, where it can be seen in globules. It is some hours before the excess is well settled. Syphon or decant the solution, leaving the excess of chloroform. It is a beautiful, clear, and sparkling preparation. Below I give a small list of formulas in which I am using it with great satisfaction, also a small list from Beurmann:

℞. Morphia sulph.................................1 grain

Aqua chloroform.............................4 fl. ℥

M. Dose, a teaspoonful every hour in irritating coughs, also in nausea, gastralgia, etc.

℞. Tinct. ferri. mur...............................4 fl. ℥

Acid phos. dil.....................................1 ʒ

Chloroform water ...............................6 ʒ

M. Dose, a teaspoonful in a half a wine glass of water before meals as a tonic.

℞. Brom. potass.....................................2 ℥
Tinct. opi. camphor..............................2 fl. ℥
Syrup tolu............................................4 fl. ℥
Chloroform water..................................1 fl. ʒ

M. Dose, from one-fourth to one teaspoonful in therapeutics of infancy.

℞. Salicylate of soda............................ 8 parts
Syrup.................................................. 30 parts
Peppermint water................................ 20 parts
*Dilute chloroform water.....................100 parts

Mix.—*Beurmann.*

℞. Chloroform water..............................13 parts
Peppermint water................................ 3 parts
Water..................................................12 parts

M. Dose, a tablespoonful for a calmative stomach potion.—*Beurmann.*

In the search after new remedies please try the aqua chloroformi.—A. D. Bundy, M.D., in *Iowa State Med. Rep.*

**How to Take a Pill.**—A writer in Med. and Surg. Reporter says: Not wishing to give a history of the pill, let me say that the pill has been rapidly gaining ground—and deservedly so. In the pill we have the exact dose always at hand, it is cleanly, and we need not trouble with spoons and bottles; it is a preserver of drugs,

*Half water.

especially in the easily soluble sugar-coated form. Further, the bad taste and disgust left behind by some medicines, as also the discoloration and bad effect on the teeth are entirely avoided.

The pill takes the medicine where it belongs, to the stomach and intestines. In my opinion, the pill will push to the wall a great many of the liquid forms in which some drugs are dispensed to-day.

A very large class of persons cannot swallow a pill of any description or make; to this class I belonged until last winter, when I had occasion to take chininum sulph., and by a simple method that Dr. E. S. Hanna told me of, I was enabled to take pills as easily as the most confirmed pill consumer.

Another class of persons succeed in taking pills but by a mighty effort, sometimes hours before taking the dose they begin to make up their minds, as we say, to swallow it without any ado, but as the hour for taking the dose draws near they think of jellies, preserves, prunes, etc., in which to more easily swallow the pill, and even then how often does it end in failure?

The modus operandi of Dr. Hanna's method which I had tried with complete success on many patients, who were taught by their former experience that it was impossible for them to swallow a pill, at least, in the ordinary way—*is to place one or more pills under the tongue.* I repeat, to place one or more pills under the tongue, then take a mouthful of water or other liquid and swallow (just as in the act of driking); this done, look for the pill. Invariably the "I-can't-take-a-pill-patient" is astonished, and sometimes he investigates the mouth with his fingers to reassure himself if he has really swallowed the pill.

The success—secret—as you wish, lies in the fact that in the act of drinking the tongue curves back upon itself, the pill, taken by the force of the current, is imperceptibly washed down the œsophagus.

I suggest that the druggists in this as in other countries will have the directions, as above, "how to take a pill," printed on every prescription label intended for pills.

Further, for this country, I would propose these directions be given in German as well as in the English language.

Further, I suggest that every editor of a newspaper, in this, as well as in other countries, under whose notice this may chance to come, will give this simple method, how to take a pill, a space in their paper, and thereby do a large class of readers a real service, which they will receive with grateful recognition.

**A New Treatment for Goitre.**—The new treatment used by Dr. A. Weiss (Berlin klin. Wochenschrift, No. 2, 1885), is the application of heated points to the surface of the tumor. By means of a Paquelin apparatus with a pointed iron, he makes punctures about one centimetre apart, the iron being at a white heat. The burns result in little scabs of one to three millimetres, which fall off in a few days, leaving behind them cicatrices which are at first red and then white. If the iron is at a white heat there is but little pain, and the consecutive treatment consists simply in covering the wound with a layer of wadding. The operation is repeated every six or eight days until the disappearance of the goitre, which requires, according to circumstances, from six to twelve applications. At the same time a little iodide of potassium is given, but this is not essential. This treatment is particularly beneficial in endemic parenchymatous goitre; in cystic goitre it is not so much so. Sometimes during treatment the goitre ceases to diminish; he then applies, immediately after the operation, a layer of vaseline over the burns; in this way suppuration is set up under the scabs, which hastens the cure. In explaining the curative action, Dr. Weiss considers that the heated points provoke an excitation of the terminal nerve filaments, which cause a contraction of the muscular coat of the vessels, resulting in an arrest of nutrition and atrophy of the hypertrophied glandular substance. In one case where the goitre was so covered by a net work of large vessels that Prof. Luke could not administer an injection of iodine or arsenic, Weiss observed, after four or five applications of the heated points, a dimunition in the calibre of these same vessels, which became normal. In this same case he applied the heated points to a greatly dilated vein in the axilla, and the next day noted a diminution in its volume. He is inclined to use this treatment in other affections; as catarrh, affections of the pleura. In one case of laryngo-tracheal catarrh, an absolute extinction of the voice of several days' standing was completely restored by one application of the heated points.—*Med. Progress.*

**Cholera Inoculation.**—The idea that a disease, one attack of which is well known not to destroy susceptibility to future attacks, can be prevented by inoculation with any possible preparation of its efficient or specific cause, is so contrary to the simple rules of induction, or in other words, to "common sense," that one feels a degree of surprise that any intelligent medical man should waste his time in experimenting on the subject. But it appears that nothing is so extravagant or absurd, especially if connected with the modern germ theories of disease, but that it may find advocates willing to experiment wherever they can find deluded subjects willing to be used for that purpose. That one attack of genuine epidemic cholera does not afford the individual immunity from subsequent attacks of the same disease is well known to all who have had practical experience with the disease. A correspondent of the British Medical Journal, of June 27, writing from Valencia, in Spain, says: "I know people at home and abroad, and have patients here, who have had it twice and thrice, and some have died of it after one attack." Yet the same writer tells us that a Dr. Ferran claims to have taken the genuine Koch bacilli from the intestines of a patient who died of cholera in Marseilles last year, and by cultivation has discovered the "basic cell of the comma-bacillus."

These basic cells he has called "peronospora Ferrani; and it is by inoculation with these that he claims to protect the people against a liability to be attacked with the cholera. The same correspondent states that in the city of Alcria alone, some 6,000 or

7,000 persons were inoculated and re-inoculated by Dr. Ferran and his assistants, who claim that it produces no severe or dangerous symptoms, and secures to the re-inoculated entire immunity from attacks of cholera. But the correspondent tells us that cases of "phlegmonious erysipelas, septicæmia, and death," following the re-inoculations, have already come under his own observation. And so far as prevention of cholera attacks is concerned, subsequent observations have shown that wherever the disease has prevailed, it has attacked the inoculated as readily and fatally as the uninoculated. It is stated in the secular papers of July 18, that "every one of forty-seven nuns who were inoculated by Dr. Ferran has since died of cholera." It has been stated several times that the Spanish government had forbidden the further operations of Dr. Ferran in that country, but it appears that he still finds deluded victims willing to submit to his futile and somewhat dangerous experiments.—*Jour. Am. Med. Association.*

**Prostatotomy**.—C. A. Harmon, M.D., in Medical Age, says: This is an operation which I believe has not been advised or attempted; yet, when we consider the great number who die from hypertrophy of the prostate and its sequelæ, there would seem to be a demand for some mode of relief.

When the hypertrophy obstructs the flow of urine, all authorities advise the use of the catheter, and the instruction of the patient in the manner of its introduction. My observation has been that when the use of the catheter becomes frequently necessary, life is drawing near its end, catheter fever or cystitis stepping in to close up the case.

This disease is the cause of much suffering and the loss of many lives of men, who, could it be cured, would be good for ten or twenty years of active business life. Ovariotomy has saved the lives of many women, why not prostatotomy save the lives of many men? The operation is simpler, the peritoneum not being opened, and drainage easy.

The first steps in the operation will be the same as in lithotomy. When the gland is reached you will proceed according to the extent to which you wish to carry the operation, but as in most subjects the gland surrounds the urethra, making it impossible to remove it all, and as the middle or false lobe is the offending part, it will only be necessary to remove that part, which can be done by making your incision down to and through the prosterior portion of the isthmus connecting the lateral lobes to a point beyond the point of entrance into the urethra of the ejaculatory ducts (very important ducts even to men of fifty or sixty years), then from the bottom of this incision make a V by two incisions, inclosing the middle lobe; then peel the lobe out of its capsule. This lobe has a very thin capsule, but it is very strongly reinforced by the bladder.

The vessels encountered in this operation are the internal pudic, vesical and hemorrodial—all small. The injury done to the vessels will, probably, by the diminished blood supply, arrest the growth of or cause the atrophy of the lateral lobes.

Living in a small city, it may be some time before I will have an opportunity to put the operation to a practical test; but it looks to me feasible, and I will claim the credit for its origination if no one else antedates me.

Years ago, to my medical associates, I spoke of the feasibility of tapping the pericardial sac and the ventricles of the brain, but made no record of the same. They have since become recognized operations.—*Medical Age.*

**The Abortive Treatment of Typhoid Fever with Naphthalin.**—L Gotze, in Reitchr. fur Klin. Med., 1885, No. 1, (Central blatt fur Klid. Med.) reports the use of naphthalin in a local epidemic of typhoid fever. Thirty-five cases in all were treated with the following results. With the exception of three cases treated with the drug, enlargement of the spleen was not noticed. The remedy was administered in doses of 15 grains, and 75, 90, and even 105 grains daily were received by the patients. Other remedies were substantially not administered.

The napthalin employed was that directed by Rossbach, the purest resublimated with the oil of bergamot. During the course of the disease all the patients received over 1,050 grains.

Untoward effects (with one exception, in which napthalian intoxication was exhibited chiefly in the form of brain depression, which was readily relieved by soda sulphurata,) were not observed.

The course of the disease, on the whole, was exceptionally favorable, and the intestinal symptoms—diarrhœa, pain in the cœcal region—was favorably influenced. In three cases the disease appeared to be generally shortened by the drug.

Other cases through its use became abortive in from six to ten days, in some others the fever did not continue more than sixteen days. In a final series of cases the process of the disease was not shortened, but the period during which marked elevation of temperature was maintained was curtailed. Three cases died from serious complications.

In cases in which antipyrin in apyretic doses failed to reduce the temperature, napthalin produced such effect, as was proven by several trials always with the same result.—*Med. News.*

**Reduced Iron in the Treatment of Anæmia**—Dr. John W. Martin, of Sheffield, England, states in the Medical Press and Circular of December 3rd, 1884, that for some time he has been using reduced iron in the treatment of anæmia with the greatest success, and thinks that it is one of the most powerful remedies which we possess in restoring the condition of the blood in all anæmic states of the system. He has employed it chiefly in chlorosis, amenorrhœa, chorea and enlargement of the spleen following intermittent fever, and states that with the exception of those cases in which the impoverishment of the blood has been due to nursing, the cases in which he has found this form of iron most serviceable are those of young girls and women of chlorotic tendencies, and in women who have reached the change of life, in whom, beyond an unexplainable failure in the powers of nutrition, no organic disease is discernible. According to him, administration of this drug has been followed by symptoms of improvement within forty-eight hours, and the patients have steadily progressed to convalescence without change of treatment. When a tendency to constipation is present this may be best overcome by the use of the following mixture:

| | | |
|---|---|---|
| ℞. | Magnesii sulph | ʒ iiss |
| | Magnesii carb | ℥ ij |
| | Tr. nucis vom | mxl |
| | Sp. amm. aromat } aa | ℥ ij |
| | Tr. cardamomi, } | |
| | Aq. menth. pip., ad | ℥ viij |
| M. | ℥ i bis die. | |

The iron is usually given in the form of a pill, of which the ingredients and the strength of their dosage may be varied as circumstances may demand. The following prescription is that which he has found most serviceable:

| | | |
|---|---|---|
| ℞. | Ferri redact | grs. iiss |
| | Ext. nucis vom | gr. $\frac{1}{8}$ |
| | Ext. quassiæ | q. s. |

M. ft. pil. no. xij.
S. One to be taken three times a day at meal times.—*Jr. Am. Med. Association.*

**Painless Removal of Toe-nail.**—Dr. F. Peyre Porcher (Med. News, July 11, 1885), reports a case in which the instillation of a four per cent. solution of hydrochlorate of cocaine, from the point of a hypodermic needle upon the slightly raw surfaces on each side of the ingrowing nail of the big toe, was perfectly successful in producing complete anæsthesia; a rag wet with the same solution being kept pressed against the upper surface of the toe and three injections of the same solution being made subcutaneously at the base of the nail. Probably not more than ten or fifteen drops in all were received and absorbed. After the lapse of fifteen minutes the narrow blade of a fine pointed pair of scissors was passed under the nail, which was divided to the matrix, when the two portions of nail were forcibly extracted without causing any suffering.

**Atonic Dyspepsia.**—Dr. Preston, at Baltimore Polyclinic, (Maryland Med. Journal), says: Various experiments have been made in the treatment of Atonic dyspepsia, which is by far the most common form of indigestion met with at the Polyclinic.

The alkaline treatment, even in cases where acidity was marked, was soon discarded as being only temporary. Sometimes the combination of a simple bitter, as tincture of columbo with soda bicarbonate, acts well for a time. Pepsin has proved of little value in adults unless given in quantities larger than most dispenaries can afford, or than a patient will take.

The most generally useful drug is strychina in the form of tinct. nux vomica. This can be given in much larger doses than it is prescribed. For many of the cases the initial dose was gtt. x to xx t. i. d. with as much acid hydrochlor. dil. This given before meals in cases where the normal acid is in excess, and after meals where it is deficient in quantity is of inestimable service. It is by no means a new treatment, but after a somewhat careful and extensive experience with it, it has proved the most satisfactory.

In some of these cases where, in addition to the ordinary symptoms, there is pain, a very good plan is to add to the above, *m* i to iii of acid hydrocyanic, dil.

This drug seems to have a peculiar sedative action upon the terminal nerves of the stomach, and will be found useful in various painful affections of this organ.

Many of these cases improve rapidly on iron, and the best way to overcome the unpleasant effects which often prevent its use is by combining gr. x of pot. brom. with gtt. x to xx of the tincture of the chloride.

**Tamponing the Nasal Fossæ with Plugs Soaked in Turpentine.**—Dr. L. Bodier, in the Jour. et de Chir. Pratique for May 1885, praises this as a simple and efficacious process which he has often employed to arrest nasal hemorrhage. This method occurred to him after a case in which he had used Belloc's sound and plugged the nares so effectually that for ten days the blood, not being able to escape anteriorly or posteriorly, welled up through the lachrymal canals; two little streams of blood running down the cheeks for about eight days. The case was one affected with slight fever, but resulted in the formation of petechiæ, intestinal hemorrhage and death. Comprehending from this that tamponing alone would not suffice, and satisfied of the insufficiency of perchloride of iron, he made a tampon of small balls of wadding attached to each other by a string like the tail of a kite (in other words, the old classic vaginal tampon), soaked the balls in turpentine, squeezed them afterwards, and filled the anterior nasal fossæ with them, without regard to the posterior fossæ, which remained open. The heat causes a part of the turpentine to vaporize, and the most inaccessible folds of the fossæ come under its irritating influence, and by this means he has treated successfully twenty or more cases under diverse circumstances.—*Journal American Medical Association.*

**Hygrine or Cocaine.**—Cocaine, the wonderful drug which anæsthetizes mucous membranes, and has simplified many minor operations on the eye, is very costly. Hence, we must not be surprised to hear that, Paris at all events, the manufacturers endeavor to obtain as much of the alkaloid as possible by submitting the coca leaves to a second process of exhaustion. The result is very similar to that which follows attempts to make a second infusion out of already exhausted tea leaves. Certain subtances are extracted which are not cocaine, but have nevertheless a remarkable effect on the pupil, causing it to dilate in a marked manner. These substances are derivatives of hygrine, most of which are mydriatics. Eserine is not an efficient antagonist to atropine, but it is to these derivatives of hygrine. Our readers may have observed that different observers have made different statements with regard to the action of cocaine on the pupil. Some have asserted that cocaine has a powerful mydriatic effect, which others have not noticed. This difference may be explained by the above mentioned facts.—*Lancet*, *May*, *1885*.

**Sulphate of Magnesia in Dropsy.**—In treating a very severe case the past spring, I found myself at the end of my list of remedies, and the patient not cured. There was a marked impairment of the heart's action, enfeebled kidneys, an enlarged spleen, and an active liver. The patient had improved some on digitalis, less on Convallaria, had been benefited by Santonine, had been injured by sulphate of manganese—a remedy that had cured him of the same trouble some years before—had exhausted the virtues of Elaterium, and now I wanted something more.

I stated the case to Prof. Locke, and received reply: "I have found that an infusion of digitalis is much better than the tincture. Of all the remedies I have employed to obtain a hydragogue action on the bowels, none has given me as much satisfaction as teaspoonful doses of sulphate of magnesia, taken in small portions of water."

The result was as described—"a tolerance of stomach and bowels for the epsom salts to hydragogue catharsis, that had not been obtained for other medicines," the infusion of digitalis having a better action upon the kidneys than the tincture. With the two we have a cure in a protracted and seemingly incurable case.

**Corrosive Sublimate in Veneral Warts.**—A correspondent writes us that having been advised to apply a solution of one grain to the ounce of corrosive sublimate, to a case of veneral warts which came under his care, he found after the application through a mistake a solution of ten grains to the ounce was applied. The result was so satisfactory that he determined to still further increase the strength, and on his next case he made the solution of twenty grains to the ounce with excellent results. He now also applies this solution to chancres and chancroids, and also to indolent ulcers of the uterus, and is highly satisfied with the results. He has never witnessed the slightest symptoms of mercurial poisoning from this treatment, and does not believe that the application of corrosive sublimate in this strength is liable to be followed by absorption.—*Medical Age*.

**Cold in the Head.**—Dr. J. L. Davis says that in a case of common cold in the head he has used the following with complete success: Half a grain of tartar emetic is dissolved in four ounces of water, and a teaspoonful of this is given every fifteen minutes for four doses, and then hourly, and after that every three or four hours. The disease is often cured in the course of one day.—*Med. World*.

**Piles and Fissured Anus.**—No pain is more atrocious than that of hemorrhoids, especially when accompanied by fissured anus, and any remedy which promises relief in such cases is hailed as a godsend by not only the patient, but by the physician who is so unfortunate to ever have had charge of a case. For this reason the thesis of M. Pauzet, recently sustained in Paris, and published in the Paris Medical, will have more attractions than these usually possess to the general medical reader. Briefly stated, it claims that 2 3-grain pills of the extract of capsicum annum, taken every morning and evening, will calm the pains and cure the disease. M. Pauzet refers to Drs. Dujardin Beaumetz, Constantin Paul, and others equally noted, for confirmation of his statement.

The following is the treatment of a case in detail, as reported in the Paris Medical: The patient, a woman, was ordered two pills of extract of capsicum annum of 3 grains each, every morning and evening. On going to bed she took a small dose of salts or a podophyllin pill, and in the morning before going to stool she injected the lower bowel with warm *eau de guimauve* (marshmallow). In five days natural functions were reestablished without pain.—*National Druggist.*

**Morphia in Cholera Infantum.**—Dr. Spencer M. Free, of Pennsylvania, writes in the American Journal of Obstetrics, claiming that true cholera infantum (not the other bowel difficulties, improperly called cholera and infantum) is so similar to sporadic cholera of adults, commonly known as cholera morbus, that the same principle of treatment was suggested to his mind.

He reports cases occuring in children from one to two years old in which he gave morphia, either hypodermically or dry upon the tongue, followed by the same prompt and beneficial effects observed after the administration of morphia in the disease in adults, namely: sleep, rest from vomiting, and diarhœa. "Nature is afforded an opportunity to recover its balance." He insists that the dose must be large enough to produce full physiological effects, and it must be given, hypodermically or dry, on the tongue, otherwise the little patient will vomit, if given in solution.

He has given as high as one-twelfth grains dry on the tongue to a child fifteen months old.—*Buffalo Med. Journal.*

**Tannic Acid Injections in Cholera.**—During the recent exceptionally severe epidemic of cholera at Naples, Prof. Cantani proposed combating the preliminary diarrhœa by warm tannic acid enemata. The practice was largely and successfully adopted. Dr. Vincenzo Vitone has just published, in Il Morgagni, for last month, his results with that method of treatment at the cholera orphanage at Antonio a Tarsia. The disease was promptly arrested in a large number of cases. The following clyster was administered: Warm water, 400 grammes; tannic acid, two grammes; gum arabic, fifteen grammes; laudanum, five drops. In adults Dr. E. Villani has injected, per rectum, as much as fifteen grammes of tannic acid in two litres of warm water, with invariably good results.—*N. Y. Med. Times.*

**Chancre.**—The rapid cure of simple non-infecting chancre has attracted much experimental inquiry among specialists. Ricord gives preference to a 15-grain solution of nitrate of silver in water. Fournier endorses Ricord's silver solution, and recommends ferric potassic tartrate, 10 parts to 100 of water and iodoform. Von Hebra, of Vienna, successfully treats chancre in four or five days by the topical use of salicylic acid. The method consists in cleansing the parts thoroughly with tepid water and soap, especially where lead, zinc, or silver has been employed, covering the sore with salicylic acid powder, and retaining it in place with adhesive strap. These dressings are renewed once or twice a day as occasion requires. The advantages claimed for this method are the prevent ion of chancrous buboes.—*Dr. Thomas, Med. Soc. California.*

**Treatment of Intermittent Fever by the Intermittent Current.**—A. Ch. Grigorjew and A. G. Musikantow (Rússkaja Medicina, 1884, Nos. 29 and 30), treated obstinate cases of intermittent fever of quotidian and tertian type by faradization of the spleen in a horizontal and vertical direction, seven and a half minutes each. They obtained out of twenty-six cases eight cures without resorting to any other medication. Twenty other cases were treated, the patients taking the electrodes in their hands. Seven of them got well without other treatment. In those cases where the spleen was locally faradized, a permanent diminution of the size of that organ was observable, even in those cases where no cure was effected.—*Alienist and Neurologist.*

**Irritable Bladder in Old Men.**—Nervous old men often suffer a martydom from a form of irritable bladder which is not dependent on organic disease. If they take a little more wine than usual, or a richer diet, or become worried and irritable in mind, they will be called upon to micturate oftener than usual and in the night. The state of urine causing this is generally dependent upon the stomach being a little "out of order," and a few doses of liquor potassæ or bicarbonate of potash, after meals, a mild sedative and a gentle purge, will often remove the trouble. Sometimes a small dose of blue pill or calomel cures him at once. If obstinate, an opium or henbane suppository is of use.

**Salt Baths in the Treatment of Fever.**—Rabinowitsch (Wratsch; Dtsch. Med. Ztg.) reports the result of the treatment in the cases of sixteen patients, who received in all one hundred and forty-one baths. He says that not only did the addition of salt to the water cause a greater reduction of temperature, but the pulse and respiration were improved and the patients felt much stronger than was the case after the use of fresh water.—*N. Y. Med. Journal.*

**Buzzing Noise from Quinine.**—It is said that "the distressing ear-symptoms produced by the administration of quinine or salicylate of soda are counteracted by the addition of small doses of ergot to the mixture."—*Medical World.*

**Piles, their Hypodermic Injection.**—Dr. J. W. Girard (Med. Bulletin) says that he has used hypodermic injections for ten years in treating piles, treating about two hundred cases without a single failure, or return of the tumor. He takes one part tannic acid, two parts carbolic acid, and eight parts of glycerin. With this mixture he injects each pile separately, and in a few days they slough away and generally heal kindly under dressings of carbolic cerate. If there be much constitutional disturbance he controls it with a steam bath or a hot sitz bath.—*Detroit Lancet.*

**Peculiar Results of Bee-Stings.**—In the Cincinnati Medical News, October, 1884, Dr. E. A. Cobleigh, of Athens, Tennessee, calls attention to some peculiar phenomena that resulted from the sting of a bee in the case of his own children. In one case, a sting on the thumb was followed by extensive urticaria on one side of the face, neck and shoulder; while, in a second case, a sting on the ball of the great toe of the right foot was followed in a few minutes by an œdema of the left side of the face, which persisted for twelve hours or more, and then gradually disappeared.—*N. Y. Med. Times.*

**Paralysis Agitans.**—Dr. Preston, at Baltimore Polyclinic, Md. Med. Journal, reports a case of paralysis agitans in a man aged 69 years, was very much relieved by a month's treatment with potas. brom. The case was of five year's standing, and was gradually getting worse; while under treatment he regained strength, and the tremor was diminished to such an extent that the man said he was not incommoded by it any longer.

It has been discovered, by experiments with dogs placed under the influence of morphia even to coma, that the hypodermic injection of a solution of theine is almost an instantaneous antidote, neutralizing the effect of the narcotic and reviving the animal after the action of the heart has become imperceptible. Caffeine possesses similar properties, but is less immediate in its operation.—*Louisville Med. News.*

**If** a saturated solution of hydrochlorate of cocaine in strong nitric acid be applied to the skin, instead of the painful smarting of the acid alone, a slight pricking sensation is the only sensory effect produced. An eschar is formed, but takes a longer time to appear than if the nitric acid be used alone. These results have been obtained by Messrs. Randolph and Dixon.—*Pharmaceutical Journal.*

**Sugar in Phthisis and Dysentery.**—Dr. Vildosola, in a Habana Medical paper, states that cane sugar is valuable as a diet in consumption and chronic bronchitis; also in dysentery, and even in dyspepsia. He says that dysentery, which cannot be controlled by ipecacuanha and other remedies is frequently found to yield to sugar-cane in a state of fermentation when chewed.—*London Lancet.*

